# Soil Behaviour of the Veterinary Drugs Lincomycin, Monensin, and Roxarsone and Their Toxicity on Environmental Organisms

**DOI:** 10.3390/molecules24244465

**Published:** 2019-12-05

**Authors:** Peiyi Li, Yizhao Wu, Yali Wang, Jiangping Qiu, Yinsheng Li

**Affiliations:** School of Agriculture and Biology, Shanghai Jiao Tong University, Shanghai 200240, China

**Keywords:** lincomycin, monensin, roxarsone, migration, residual, toxicity

## Abstract

Lincomycin, monensin, and roxarsone are commonly used veterinary drugs. This study investigated their behaviours in different soils and their toxic effects on environmental organisms. Sorption and mobility analyses were performed to detect the migration capacity of drugs in soils. Toxic effects were evaluated by inhibition or acute toxicity tests on six organism species: algae, plants, daphnia, fish, earthworms and quails. The log K_d_ values (Freundlich model) of drugs were: lincomycin in laterite soil was 1.82; monensin in laterite soil was 2.76; and roxarsone in black soil was 1.29. The R_f_ value of lincomycin, roxarsone, monensin were 0.4995, 0.4493 and 0.8348 in laterite soil, and 0.5258, 0.5835 and 0.8033 in black soil, respectively. The EC_50_ for *Scenedesmus obliquus*, *Arabidopsis thaliana*, *Daphnia magna* and LC_50_/LD_50_ for *Eisenia fetida*, *Danio rerio*, and *Coturnix coturnix* were: 13.15 mg/L,32.18 mg/kg dry soil,292.6 mg/L,452.7 mg/L,5.74 g/kg dry soil and 103.9 mg/kg (roxarsone); 1.085 mg/L, <25 mg/kg dry soil, 21.1 mg/L, 4.76 mg/L, 0.346 g/kg dry soil and 672.8 mg/kg (monensin); 0.813 mg/L, 35.40 mg/kg dry soil, >400 mg/L, >2800 mg/L, >15 g/kg dry soil, >2000 mg/kg (lincomycin). These results showed that the environmental effects of veterinary drug residues should not be neglected, due to their mobility in environmental media and potential toxic effects on environmental organisms.

## 1. Introduction

The environmental effects of drug residues have received increasing attention as a new type of pollutant. In addition to their therapeutic purposes, veterinary drugs are also incorporated into animal feed as additives to improve animal growth rate and feed efficiency [1]. In the United States, 112,000 tons antibiotics were used for cattle and pig livestock every year for non-therapeutic purposes [2]. Many studies have indicated that only a small fraction of the veterinary drugs consumed by livestock and pets are metabolized; the majority are released into the environment in their original forms, and can potentially enter the food chain to pose human health risks [3,4,5]. Drug residues can exist widely in the environment, inducing antibiotics, which can lead to increased drug resistances and reduced effectiveness in human and veterinary medicine [6]. One study found that more than 500 different types of veterinary drugs and more than 100 metabolites in the aquatic environment of 71 countries covering all continents, and the concentrations of some drugs, such as diclofenac, were measured at higher than the safe dose level in some countries [7]. Previous research on the occurrence of 13 veterinary drugs in 23 vegetable fields in eastern China where animal manure was used found that animal feces, especially those from poultry farms, was an important source of veterinary drug accumulation in soil [8]. It has also been shown that people can excrete carbamazepine and its metabolites after consuming food from crops irrigated by waste water, suggesting that human beings can ingest drugs from residue pollution [9]. Therefore, drugs can accumulate in soil, be absorbed by plants, and may have harmful effects on organisms.

Lincomycin is a commonly used lincosamide antibiotic for veterinary purposes, especially in China. It is persistent in the environment due to its pyranose ring, amide, pyrrolidine ring and other structures [10], and exhibits inhibitory effects and toxicity in organisms [11]. Lincomycin has numerous detrimental effects on the haematological and biochemical properties of blood, and can interfere with liver and kidney functions [12]. The EC_50_ value of lincomycin for *Artemia* (brine shrimp) was 283.1 mg/L [13], and the IC_50_ values of lincomycin for *Cylindrotheca closterium* and *Navicula ramosissima* were 14.16 mg/L and 11.08 mg/L, respectively [14]. The toxic effect of lincomycin, tylosin and ciprofloxacin mixture were found synergistic against *C. closterium* and additive for *N. ramosissima* [14]. Recently, the occurrence of antibiotic resistance has heightened concerns over lincomycin usage [15].

Monensin, the most widely used coccidiostat in the U.S. [16], is a class of polyether ion-carrier antibiotic used for the livestock and poultry industry. Monensin in fresh chicken manure can pose an environmental risk under certain conditions, and the use of compost was shown to be a method to degrade monensin [17]. The half-life of monensin ranges from 4–15 d in high-intensity management (i.e., soil amending, watering, and turning) and from 8–30 d in low-intensity management [18]. Because of its widespread use and high persistence, monensin was detected in multiple environments: 0.3 ± 4.5 mg/L in manure; 0.0004 µg/kg in soil; 0.01 ± 0.05 µg/L in surface water; 0.04 ± 0.39 µg/L in underground water; and 1.5 ± 31.5 µg/kg in sediment [16]. When the monensin concentration was 0.05 µmol/L, the adsorption coefficient of various soils was in the range of 0.915–78.6 L/kg [19]. Based on the K_d_ value, monensin is more mobile than tetracycline and has similar mobility to sulfamethazine. Its toxicity is highly species dependent [19]. It was reported that 50 mg/kg monensin could inhibit the reproduction and survival of earthworms [20]. Due to its high usage, high toxicity characteristics, and unevaluated potential environmental impacts, monensin has been classified as a high-priority environmental pollutant requiring further assessment [21].

Roxarsone is commonly added to the feed for farmed broiler chickens, and nearly all roxarsone is excreted unchanged in the manure [22]. Roxarsone can significantly induce CYP1A2 activity in the pig liver microsome, and the induction effect of it was stronger than enrofloxacin [23]. Zhang [24] found that the average elimination half-life of roxarsone in soil was 26.6–44.9 d and its adsorption in different soil depths was consistent with the Fetter linear adsorption model. Makris et al. [25] found that roxarsone had a higher adsorption capacity than inorganic arsenic (IV) in soil, which may be caused by the organic properties of roxarsone. After entering the soil and water system, roxarsone can be transformed into inorganic arsenic (III) and (V) with stronger migration capacity and greater toxicity [26], and can affect the growth and development of various plants. However, further studies are required to more thoroughly assess the potential environmental effects of roxarsone.

This study focused on the migration of lincomycin, monensin, and roxarsone in several soil environments, and their toxic effects on representative environmental organisms: *Scenedesmus obliquus* (algae), *Arabidopsis thaliana* (plant), *Eisenia fetida* (earthworm), *Danio rerio* (zebrafish), *Daphnia magna* (crustacean) and *Coturnix coturnix* (quail). The aim was to evaluate the environmental risks of the three drugs and provide a foundation for an impact assessment of their environmental residues for pollution management and prevention. 

## 2. Results & Discussion

### 2.1. Adsorption-Desorption Test

The adsorption rates are shown in Table 1, and the parameters of the Freundlich model fitted are shown in Table 2. The log K_d_ value of lincomycin and monensin in laterite soil were 1.82 and 2.76. The log K_d_ value of roxarsone in black soil was 1.29. The corresponding 1/n values of the three equations were less than 2. According to the K_d_ value, the adsorption of laterite to monensin was greater than to lincomycin, which could be due to the higher molecular weight and lower water solubility of monensin. The 1/n value of lincomycin was 0.0935 in laterite soil. Therefore, the isotherm adsorption line of lincomycin belonged to the “l” type, meaning that at a certain concentration range, the adsorption capacity of soil to lincomycin decreases with increasing drug concentration. This result was consistent with the observed trend of the lincomycin adsorption rate. Another study showed that when the initial drug concentration was 1 mg/L, the adsorption rate of ethanamizuril in podzol soil was approx. 70% and decreased with increasing drug concentration, indicating that podzol soil displayed a stronger adsorption to lincomycin and monensin than ethanamizuril [27]. The lower organic matter contents and higher pH of the laterite and podzol soil are properties that may have contributed the smaller adsorption of roxarsone (pKa_1_ = 3.49). Rutherford et al. [28] found that when 2 < pH < 8, the adsorption of roxarsone in soils decreased as pH increased. When the pH of the soil solution increased, the negative charge of the soil surface increased, causing an increased repulsive force between roxarsone and soil colloid, and a lower soil adsorption capacity of roxarsone. The zero adsorption of roxarsone was observed in field soil (pH 8.4, organic matter 6.84%) and wasteland soil (pH 8.2, organic matter 4.73%) [29].

### 2.2. Soil Mobility Test

Soil mobility test results are shown in Table 3. In laterite soil, the ranking of mobility capacity of drugs was monensin > lincomycin > roxarsone, and in black soil, the ranking was monensin > roxarsone > lincomycin. According to the classification in GB/T 31270-2014 (Test guidelines on environmental safety assessment for chemical pesticides) [30], lincomycin and roxarsone were moderately mobile in laterite and black soils, while monensin was highly mobile. Generally, adsorption ability has a negative correlation with water solubility of drugs, and a positive correlation with soil organic content; migration ability has the opposite correlations [31]. A previous study showed that the R_f_ value of roxarsone was 0.66 in soil (pH 6.5, organic matter 19.08 g/kg), indicating strong mobility [32]. However, in the present study, the R_f_ value was 0.4993 in laterite soil (pH 6.710, organic matter 3.92 g/kg), and 0.5835 in black soil (pH 5.257, organic matter 263.69 g/kg). These differences may have resulted from other physicochemical properties [33]. Florasulam was shown to be primarily distributed in a 9–18 cm soil layer in soil (organic matter 23.57 g/kg), and its R_f_ value was 0.918 [34]. According to the R_f_ values in the presents study, the migration ability of the three drugs tested could be weaker than florasulam in a similar soil environment.

### 2.3. Algae Growth Inhibition Test

**Figure 1:**
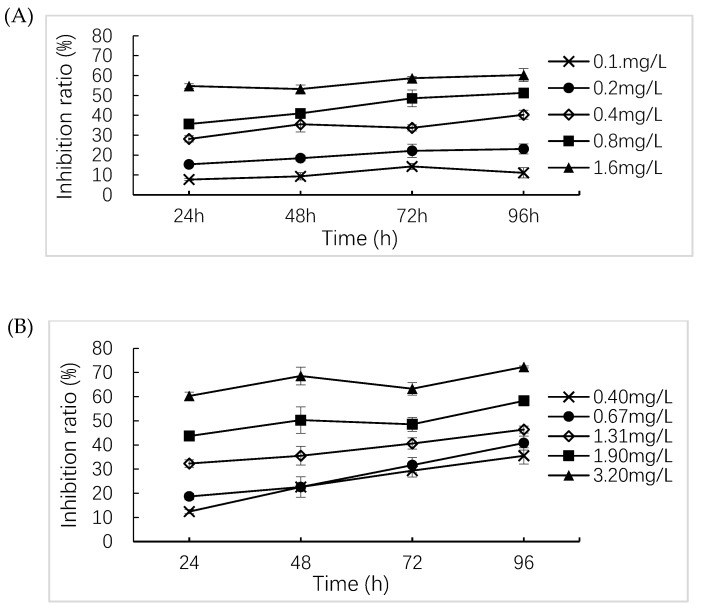

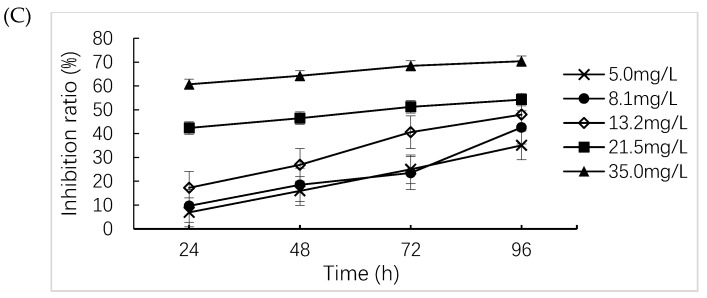
Effect of drugs on the growth of *S. obliquus.* (**A**) lincomycin, (**B**) monensin, (**C**) roxarsone.

The absorbance of algae culture remained unchanged or even decreased with time, indicating that the concentrations of the algae decreased and their growth was restrained. Figure 1A–C show that the inhibition rate of algae (*S. obliquus*) growth by the three drugs was positively correlated with drug concentration. The pH of the treatment groups and controls were 6.9–7.4 at the end of the algae growth inhibition test. As the culture time increased, the percentage inhibition of algae growth rate increased. Based on the 96-h inhibition rate, the calculated EC_50_ (96h) were 0.813 mg/L (lincomycin, 95% confidence interval [0.791, 1.489] mg/L), 1.085 mg/L (monensin, [0.554, 1.193] mg/L) and 13.15 mg/L (roxarsone, [9.70, 17.83] mg/L). These results suggested that lincomycin, monensin and roxarsone showed low, medium, and low toxicity levels to algae, respectively. The EC_50_ (96h) of lincomycin and monensin suggested that these two drugs demonstrated higher toxicity to algae than ofloxacin (6.20), sulfamethoxazole (7.20) and sulfamethazine (9.89) [35], which was consistent with the results of Peng et al. [36]. 

Peng et al. also found that lincomycin and ofloxacin presented a high ecological risk to algae; the risk quotient (RQ) of lincomycin was 1.93, followed by ofloxacin (1.33), tetracycline (0.41) and erythromycin (0.20). According to another study, lincomycin can inhibit the synthesis of the D1 protein in the algal photosynthesis system, and may lead to algal death [37].

### 2.4. Plant Sensitivity Test

The effects of drugs on plant growth are shown in Table 4 and Table 5. The seedling rates of blank controls and methanol controls (for monensin) were all above 50%, suggesting that experiments were valid.

#### 2.4.1. Lincomycin

The EC_50_ (14 d) of lincomycin on *A. thaliana* seedling rate was 35.40 mg/kg dry soil (95% confidence interval [19.217, 65.211] mg/kg dry soil), however, there was no significant difference in plant emergence on 14 d (Table 4). With increased lincomycin concentration, the number of seedlings, biomass and plant height were not significantly affected (Table 4 and Table 5).

#### 2.4.2. Monensin

The seedling rates of the blank control group and methanol solvent control group on 7 d and 14 d were both higher than 50% (Table 4). However, in the experimental groups, only 1 seedling appeared in the 25 mg/L monensin group. On 14 d, only a few seeds sprouted, and the plant heights were all less than 0.5 cm. Therefore, the EC_50_ (14 d) of monensin on *A. thaliana* seedling rate was <25 mg/kg dry soil (the minimum test concentration). The results indicated that monensin may have strong toxicity on plant growth. A study by Hoagland [38] also confirmed the high phytotoxicity of monensin: it caused herbicidal injury to 1–2 week-old seedlings of seven weed and two crop species when applied at 10^−4^ M as a foliar spray in the greenhouse, and all nine species died within 24–72 h after treatment.

#### 2.4.3. Roxarsone

The EC_50_ (14 d) of roxarsone on *A. thaliana* seedling rate was 32.18 mg/kg dry soil (95% confidence interval [11.319, 49.795] mg/kg dry soil). On 14 d, the number of plants that emerged in the blank control group was significantly higher than in each treatment group (Table 4). In the treatment groups, only the 5 mg/kg and the 80 mg/kg roxarsone groups showed significant differences between each other in seedling rate on 14 d. This indicated that roxarsone could reduce the growth of *A. thaliana*.

### 2.5. Daphnia Activity Inhibition Test 

In the control group, the daphnia (*D. magna*) body structures were clearly observed, not immobilized, and did not display unusual behaviours. However, in the high drug concentration groups, the body structure of daphnia appeared blurred, and daphnia immobilization was observed. Table 6 shows the inhibition rate of monensin and roxarsone on daphnia activity. The EC_50_ (48 h) values were 21.1 mg/L (monensin, 95% confidence interval [17.9, 24.7] mg/L) and 292.6 mg/L (roxarsone, [283.7, 301.8] mg/L), which suggested that both drugs had low toxicity on daphnia. Acute activity inhibition did not occur in the lincomycin group at all concentrations tested after 48 h. Therefore, the EC_50_ of lincomycin for daphnia was > 400 mg/L (the maximum test concentration).

### 2.6. Acute Toxicity Tests of Zebrafish

The potassium dichromate toxicity test (reference poison) for zebrafish (*D. rerio*) is shown in Table 7. The LC_50_ of potassium dichromate for zebrafish was 356.6 mg/L (95% confidence interval [333.0, 381.8] mg/L). An LC_50_ value between 200–400 mg/L demonstrated that the experimental method and the fish quality met the appropriate standards [39].

Table 8 shows the toxicity of monensin and roxarsone on zebrafish. There was no significant difference between the methanol control group and the blank control group (*p* > 0.05). The LC_50_ (96 h) values were 4.76 mg/L (monensin, 95% confidence interval [4.670, 4.853] mg/L) and 452.7 mg/L (roxarsone, [447.9, 457.5] mg/L), suggesting that monensin and roxarsone had medium and low toxicity, respectively, for zebrafish. The zebrafish in the lincomycin group did not show any abnormal activity at all concentrations tested after 96 h. Therefore, the LC_50_ (96 h) of lincomycin was >2800 mg/L (the maximum test concentration). Previous research showed that roxarsone could cause rapid DNA destruction of *Carassius auratus*, and low doses of roxarsone could cause more sustainable and increased damage than high doses [40]. The experimental LC_50_ (96 h) values of oxytetracycline and norfloxacin for zebrafish were 1.262 × 10^−3^ mol/L and 2.026 × 10^−3^ mol/L, respectively [41], suggesting their toxicity may be similar to roxarsone and lower than monensin. 

### 2.7. Acute Toxicity Tests of Earthworm

Table 9 shows the toxicity of monensin and roxarsone on earthworms (*E. fetida*). The mortality rate of the control group was less than 10%, suggesting the test was valid. The appearance of earthworms in the low concentration groups showed no difference compared to the control group. However, in the 20 g/kg roxarsone group, the earthworms atrophied, the girdles were swollen, and some earthworms died. 

There was no significant difference between the methanol control group for monensin and its blank control group (*p* > 0.05). The LC_50_ (14 d) values were 346.0 mg/kg dry soil (monensin, 95% confidence interval [309, 387] mg/kg dry soil) and 5.74 g/kg dry soil (roxarsone, [5.14, 6.41] g/kg dry soil), indicating that both drugs had low toxicity for earthworms [30]. No earthworm mortality or abnormal activity was discovered at all concentrations of lincomycin tested after 14 d. Therefore, the LC_50_ (14 d) of lincomycin was >15 g/kg dry soil (the maximum test concentration). The LC_50_ of monensin for earthworms was higher than the reported 75.883 mg/kg observed in an artificial soil test [20].

### 2.8. Acute Toxicity Test on French Giant Quail

Table 10 shows the toxicity of monensin and roxarsone on French giant quails. The mortality rate of the control group was less than 10%, suggesting the test was valid. At high concentrations of roxarsone and monensin, inappetence, listlessness and other symptoms were observed in quails. There was no significant difference between the carboxymethylcellulose sodium control and the blank control (*p* > 0.05), and no significant difference between males and females (*p* > 0.05). The LD_50_ (7 d) values were 672.8 mg/kg (monensin, 95% confidence interval [451.0, 1003.8] mg/kg) and 103.9 mg/kg (roxarsone, [80.5, 134.2] mg/kg), indicating that monensin and roxarsone displayed low and medium toxicity, respectively. All quails exposed to lincomycin survived after 7 d, and their appetite, activity, and excretion were no different from quails in the control group. Therefore, the LD_50_ (7 d) of lincomycin was > 2000 mg/kg (the maximum concentration). Another study on the effect of roxarsone on laying hens suggested that roxarsone could cause an increase in aspartate aminotransferase (AST), lactate dehydrogenase (LDH), and creatine kinase (CK) activity, a decrease in the liver weight, and histological evidence of liver damage [42]. Therefore, it is possible that roxarsone causes quail mortality via oxidative stress.

### 2.9. Summary of Results

Acute toxicity test data and toxicity category of the three drugs to six species are summarised in Table 11. Lincomycin showed low toxicity for all tested organisms, and medium toxicity for *S. obliquus*. Monensin showed medium toxicity for *S. obliquus* and *D. rerio*, and low toxicity for *E. fetida*, *D. magna*, and *C. coturnix*, and no detectable toxicity for *A. thaliana*. Roxarsone showed medium toxicity for *C. coturnix* and low toxicity for all other tested organisms.

As an important component of aquatic ecosystems, algal photosynthesis accounts for a large proportion (up to 50%) of global primary productivity [43]. Due to its toxicity on algae, the clinical dosage and release of lincomycin should be carefully regulated. Monensin showed a significantly higher toxicity risk on some species compared with other commonly used antibiotics, such as tetracyclines and quinolones. This result was consistent with previous research findings showing that ionophores exhibited higher toxicity than other antibiotics [19]. More attention should be given to the use of monensin, its residue and accumulation in the environment. Roxarsone-contaminated soil and its accumulation in rice could present serious problems for human health [44]. Despite the low ecotoxicity of roxarsone found in this study, its use, and the use of other arsenic-containing drugs, requires strict control to avoid arsenic entering the food chain. Possible methods to reduce the concentration of drugs in the environment include the treatment of animal manure before field application, the use of alternative bio-agents for disease treatment, and a well targeted legalized use of antibiotics [45]. 

## 3. Materials and Methods 

### 3.1. Chemicals, Test Soils and Organisms

Roxarsone (purity >98%) was purchased from Shanghai Titan Technology (Shanghai, China), LTD. Sodium morenate (purity >90%) and lincomycin hydrochloride (purity >95%) were purchased from Sangon Biotech LTD (Shanghai, China). The podzol soil was collected from the Shanghai suburban district. The black soil was taken from a forest in Changbai Mountain, Jilin Province, China. The laterite soil was collected from idle farmland in Chuzhou, Anhui Province, China. All test soils were collected from the surface layer (0–10 cm) that had not been farmed for more than 20 years, and were air-dried, grinded and sieved to a 2-mm size. The basic properties of the test soils are shown in Table 12.

*Scenedesmus obliquus* (green algae) were provided by the Chinese Academy of Sciences Institute of Hydrobiology (Wuhan, Hubei Province, China), and were grown in BG-11 cultures with continuous passage for three times and were tested in the logarithmic growth phase. *Arabidopsis thaliana*, wild type, were provided by School of Life Science and Biotechnology, Shanghai Jiaotong University (Shanghai, China). All seeds were treated at 4 °C for more than a week before sowing. *Daphnia magna* was provided by Guangdong Laboratory Animals Monitoring Institute (Guangzhou, Guangdong Province, China). The daphnia was domesticated in laboratory conditions for 7 d. Healthy infant daphnia were selected for experiments. *Danio rerio* was purchased from a pet house in Minhang District, Shanghai, China. The fish were domesticated in laboratory conditions for 7 d and 2 cm-long healthy fish were selected. *Eisenia fetida* were purchased from Wangjun Earthworm Farm in Jiangsu Province, China, and domesticated for 14 d. Healthy adult earthworms weighing 300–600 mg with an obvious girdle band were selected. *Coturnix coturnix* (French giant quails) were purchased from Shanghai Fengxian Quail Farm in Fengxian District, Shanghai, China. They were aged approx. 30 d and weighed approx. 100 g. The quails were domesticated for 7 d under laboratory conditions, and those with no disease were selected. All experimental methods performed were in accordance with National Standards of China [30,39,46] and OECD [47].

### 3.2. Adsorption-Desorption and Soil Mobility

#### 3.2.1. Adsorption-Desorption Test

The sorption of drugs by soils was investigated using the oscillation balance method following GB/T 21851-2008 [46]. The optimal soil/solution ratios and adsorption-desorption equilibrium time used in the formal test were obtained by a pre-experiment (Table 13). 

Soil samples (0.5–5 g) were weighed and placed in 50 mL centrifuge tubes covered with tin foil. Appropriate amounts of aqueous solution of different drug concentrations (0, 1, 2, 3, 4, and 5 mg/L) were added to the tubes to reach the optimum soil/solution ratios. The tubes were shaken at 25 ± 2 °C at 180 r/min. Three replicates were used for each concentration group. 

After the adsorption reached equilibrium, drugs in solution and soils of all groups were extracted and detected using methods described in 3.2.3 and 3.2.4. Adsorption rates were calculated and the Freundlich model (lg C_s_ = lg K_d_ + 1/n lg C_e_) was applied to fit sorption isotherms. 

#### 3.2.2. Soil Mobility Test

Mobility was investigated by soil thin-layer chromatography following GB/T 31270-2014 [30]. 10 µg of each drug was spotted 2.0 cm from the lower end of the coated soil sheet plates (containing 10 g of soil, 0.5 mm soil thickness, air-dried). Blank controls and three replicates were used. After the solvent had evaporated, the glass plate was placed at an angle of 30° in a chromatographic bath, and was then unfolded by distilled water at room temperature until the water surface reached the leading edge of the glass plate. The soil layer was divided into 6 × 3 cm segments. Each soil segment was scraped into a 10 mL centrifuge tube to detect drug content (3.2.3 and 3.2.4). The R_f_ values of drugs were calculated.

#### 3.2.3. Soil Drug Extraction

Drugs were extracted from soils according to [48]. Briefly, 10 mL of methanol was added to the soil in centrifuge tubes. The tubes were then vortexed for 1 min, sonicated at 25 °C for 10 min, and centrifuged at 8000 r/min for 5 min. The supernatant was passed through a 0.45 µm filter to obtain the pre-treated sample solution. Extraction accuracy was checked by a recovery test (Table 14). Addition concentrations were 1, 2, 4 mg/L, and five replicates for each concentration were tested in each soil.

Drug extraction from water: an appropriate volume of testing water was passed through a 0.22 µm filter to obtain the pre-treated sample solution.

#### 3.2.4. Drug Detection

Pre-treated samples from 3.2.3 were analysed using an ultraviolet spectrophotometer (Agilent, Beijing, China) and the method’s accuracy was tested.

##### Lincomycin

Lincomycin and PdCl_2_ can form a coloured complex with a maximum absorption peak at 380 nm [49] under acidic conditions. 1.6 mL of 0.02 mol/L PdCl_2_ solution was added to a pre-treated sample solution in a 50 mL volumetric flask, diluted with 1 mol/L hydrochloric acid solution, and mixed in the dark for 30 min. The absorbance was measured at 380 nm. The linear regression equation was: y (absorbance) = 0.0024 × (drug concentration) − 0.0003, R^2^ = 0.9986.

##### Monensin

A 3% vanillin solution was used as a derivatization reagent to react with monensin, and the product has a maximum absorption peak at 525 nm [50]. 2 mL of 3% vanillin solution and methanol were added to the pre-treated sample solution in a 50 mL volumetric flask, shaken, diluted, and then heated in a 45 °C water bath for 20 min. After the solution cooled to room temperature, its absorbance was measured at 525 nm. The linear regression equation was: y = 0.0329x + 0.0008, R^2^ = 0.9973.

##### Roxarsone

Roxarsone has ultraviolet-absorbing groups such as a benzene ring, and its solution has a maximum absorption peak at 264 nm [51]. The pre-treated sample solution was made up to 50 mL with distilled water and the absorbance was measured at 525 nm. The linear regression equation was: y = 0.0227x, R^2^ = 0. 9981, average RSD% = 9.77.

### 3.3. Ecotoxicology Tests

#### 3.3.1. Algae Growth Inhibition Test

*S. obliquus* was cultured in 100 mL Erlenmeyer flasks filled with 100 mL culture. The BG-11 culture was prepared according to Deng. et al. [52], with the initial pH adjusted to 7.1–7.5 with 1 M NaOH and HCl solutions. Flasks were placed in an artificial climate chamber with 12 h light and 12 h darkness at 29 °C. Light intensity was 5000 lux. 

The original algae concentration was measured by a blood cell counting plate, and sequentially diluted 1:2, 1:10, 1:50, and 1:100. The absorbance of the five gradient concentrations of the algal liquid was measured at OD 660 nm by spectrophotometry, and the equation obtained was y (absorbance) = 687.19 × 10^4^ × (algae concentration), R^2^ = 0.9982. 

Before inoculation, the absorbance was measured and the initial concentration of algae in the conical flask was diluted to 10^5^/mL according to the absorbance. The absorbance was measured again after inoculation. According to the results of the pre-experiment, the concentration of algae growth inhibition rate exceeding 50% was selected as the highest concentration of the formal test. Five concentrations and blank controls were used for each drug with three replicates (the solvent control for monensin was 0.1 mL/L dimethyl sulfoxide). The following drug concentrations were used: roxarsone: 5.0, 8.1, 13.2, 21.5, 35.0 mg/L; monensin: 0.40, 0.67, 1.31, 1.90, 3.20; lincomycin: 0.10, 0.20, 0.40, 0.80, 1.60. Monensin was solubilized with dimethyl sulfoxide at 0.1 mL/L. The absorbance of the algae solution was measured after shaking for 0 h, 24 h, 48 h, 72 h and 96 h. The pH was measured after shaking for 96 h in accordance with GB/T 21805-2008 [46].

#### 3.3.2. Plant Sensitivity Test 

The plant sensitivity test was conducted by soil culture using circular pots with a diameter of 15 cm. A 600 g sample of test soil was used at a thickness of approx. 10 cm, in accordance with GB/T 31270-2014 [30]. Lincomycin and roxarsone were dissolved in water. Monensin was solubilized with methanol at 2 mL/kg dry soil.

According to the results of the pre-experiment, five concentrations with three replicates and blank controls were used for each drug in the formal tests (the solvent control for monensin was methanol, 2 mL/kg dry soil). The following drug concentrations were used: lincomycin: 6, 12, 18, 24, 30 mg/kg dry soil; roxarsone: 5, 25, 45, 65, 80 mg/kg dry soil; and monensin: 25, 50, 75, 100, 125 mg/kg dry soil.

After the soil and the test solution were thoroughly mixed (methanol volatilized completely), 10 seeds of *A. thaliana* were evenly planted on the surface of podzol soil with conditions of 16 h light and 8 h darkness, 25 ± 2 °C (day) and 22 ± 2 °C (night), 75% humidity and 15,000 lux light intensity. The soil moisture was approx. 60% during the test. Drug effects were evaluated 14 d after emergence of the seedlings reached 50% in the control group. As the test endpoint, the seedling emergence rate of each group was calculated on 14 d after sowing. The biomass and plant height were measured on 14 d. 

#### 3.3.3. Growth Inhibition Test of Daphnia Activity

Eight *D. magna* organisms were cultured in a 100 mL Erlenmeyer flask per treatment, with 50 mL of experimental water (tap water, naturally placed for 2 d). Young daphnids (aged less than 24 h, not first brood progeny) were selected, according to GB/T 21830-2008 [46]. Specimens were incubated at 20 °C in complete darkness. Daphnids were not fed during the experiment and were moved with droppers. Monensin was solubilized with methanol to 0.1 mL/L.

According to the results of the pre-experiment, five concentrations with three replicates and blank controls were used for each drug (the solvent control for monensin was 0.1 mL/L methanol). The following drug concentrations were used: roxarsone: 250.0, 268.9, 289.3, 311.1, 334.7 mg/L; monensin: 9.5, 14.7, 20.0, 22.7, 35.0 mg/L; and lincomycin: 80, 120, 180, 270, 400 mg/L. After 24 h and 48 h, the number of immobilised daphnids (used as endpoint) were counted, and their behaviour and appearance in each conical flask were examined.

#### 3.3.4. Acute Toxicity Test of Zebrafish

Acute toxicity test of zebrafish was performed according to GB/T 27861-2011 [39]. Each aquarium (0.2 m × 0.2 m × 0.2 m) was filled with 3 L of experimental water (tap water, naturally placed for 2 d) and 8 fish.

Potassium dichromate was used for the reference poison test, and concentrations used were 0, 250, 300, 350, 400, and 450 mg/L. Mortality rate was calculated after 24 h. 

According to the pre-experiment, the lowest total lethal concentration and the highest total survival concentration of the drugs were obtained. In the formal experiment, five drug concentrations were used in that range according to the equal ratio series: roxarsone: 316, 354, 397, 445 and 500 mg/L; monensin: 4.00, 4.34, 4.70, 5.10, 5.53 mg/L. Monensin was solubilized with methanol at 0.1 mL/L. Blank solvent controls (0.1 mL/L methanol for monensin) were used. No replicates were used for each concentration group or controls.

The experimental conditions were 25 ± 2 °C, no aeration, with feeding during the experiment. Dead fish were removed to prevent water pollution. The experiment lasted 96 h, and the number of deaths and symptoms of poisoning were observed every 24 h. Temperature, oxygen concentration and pH were monitored daily. All procedures were approved by the Animal Ethical and Welfare Committee of Shanghai Jiaotong University Animal Department (Approval ID 20180301005).

#### 3.3.5. Acute Toxicity Test of Earthworms 

A 500 g sample of podzol soil, 125 mL of distilled water and drug solution were added to each experimental container. Monensin was ventilated for 24 h after mixing to volatilize solvent methanol. Ten earthworms dried by filter paper were added, with their intestines cleaned for 24 h before the experiment. 

The lowest total lethal concentration and the highest total survival concentration of the drugs were obtained in pre-experiments. In the formal experiments, five concentrations were used in that range according to the equal ratio series: roxarsone: 1.25, 2.50, 4.00, 10.00 and 20.00 g/kg dry soil; and monensin: 30, 60, 200, 350, and 550 mg/kg dry soil. Three replicates for each concentration, blank controls and solvent control (0.1 mL/L methanol for monensin) were used. 

The experimental conditions were 20 ± 2 °C, 75% humidity, and approx. 60% soil moisture. The number of deaths and poisoning symptoms were recorded on 7 d and 14 d, and mortality rate was calculated.

#### 3.3.6. Acute Toxicity Test of Quails

The experiment was performed by a one-off oral toxicity test with oral needles and catheters, in accordance with GB/T 31270-2014 [30]. The dose was 1 mL/quail, where lincomycin and roxarsone of low concentration were dissolved in water, and roxarsone of high concentration and monensin were dissolved in 0.05% sodium carboxymethylcellulose solution. 

According to the pre-experiment, the lowest total lethal concentration and the highest total survival concentration of the drugs were obtained. In the formal experiment, five concentrations were used in that range according to the equal ratio series: roxarsone: 30.0, 52.7, 100.0, 162.3 and 284.8 mg/kg; monensin: 80, 400, 800, 1100, and 1500 mg/kg. Blank controls and solvent controls (0.05% sodium carboxymethyl cellulose solution) were used. There were no replicates for each concentration group or controls.

Ten quails (5 male and 5 female) were placed in each cage, and the experimental period was 7 d. The death and symptoms of quails were observed every 24 h. All procedures were approved by the Animal Ethical and Welfare Committee of Shanghai Jiaotong University Animal Department (Approval ID 20180102008).

### 3.4. Statistical Analysis

The data were calculated using Excel 2016 (Microsoft Inc., Redmond, WA, USA). Univariate analysis of variance was performed using IBM SPSS Statistics 19.0 (International Business Machines Corporation, Armonk, NY, USA), and Probit regression model was established to calculate EC_50_, LC_50_, LD_50_ and 95% confidence intervals. The Duncan method was used to analyse significant differences at *p* < 0.05 of each group.

## 4. Conclusions

This study evaluated the adsorption and migration capacity of lincomycin, monensin and roxarsone in different soil environments and their toxic effects on diverse environmental organisms. Moderate soil mobility and high water solubility exhibited by lincomycin could assist drug transfer and accumulation in various environments, and may cause certain problems after enrichment. Roxarsone was moderately mobile and its ecotoxicity implied that it is a potential ecological risk. Monensin was the most toxic among the three drugs tested, and its higher affinity to soil made it easier to be accumulated. The potential environmental impacts identified by the drugs tested as a result of their mobility, persistence and ecotoxicity will help to inform veterinary drug management and drug residue pollution concerns. 

## Figures and Tables

**Table 1:** Adsorption rates of drugs in different soils (%).

Drug	Soil	Initial Drug Concentration (mg/L)				
		1	2	3	4	5
Lincomycin	Laterite soil	66.67	30.56	35.19	19.44	0
	Podzol soil	97.22	54.17	54.63	0	0
	Black soil	100.0	100.0	86.11	16.67	1.111
Monensin	Laterite soil	56.16	93.68	55.26	90.92	93.66
	Podzol soil	100.0	92.60	87.50	89.99	88.25
	Black soil	100.0	93.21	94.12	95.19	98.78
Roxarsone	Laterite soil	0	0	0	0	0
	Podzol soil	0	0.8080	0	0	0
	Black soil	18.80	38.18	97.41	51.95	42.00

**Table 2:** Parameters of the Freundlich model.

Drug	Soil	Lg K_d_	1/n	R^2^
Lincomycin	Podzol soil	1.5672	0.1116	0.2043
	Laterite soil	1.8235	0.0935	0.5282
	Black soil	1.7091	−1.2543	0.7329
Monensin	Podzol soil	1.5194	−0.1842	0.0355
	Laterite soil	2.7601	0.6117	0.8910
	Black soil	1.8502	−0.3915	0.2411
Roxarsone	Black soil	1.2855	1.9393	0.8759

**Table 3:** Content distribution of drugs using soil thin layer chromatography.

Drug	Soil	Drug Content in Each Moving Distance (%)						R_f_
		0–3 cm	3–6 cm	6–9 cm	9–12 cm	12–15 cm	15–18 cm	
Lincomycin	Laterite soil	9.250	27.74	19.99	10.54	12.09	20.40	0.4995
	Black soil	16.11	15.63	15.01	14.66	17.07	21.51	0.5258
Monensin	Laterite soil	8.154	0	0	4.154	0	87.69	0.8348
	Black soil	0	0	0	0	68.02	31.98	0.8033
Roxarsone	Laterite soil	17.51	20.93	17.96	20.98	13.31	9.310	0.4493
	Black soil	12.45	12.99	10.85	13.10	26.86	23.74	0.5835

**Table 4:** Number of plants emerged in drug groups (n = 10 seeds).

**Monensin**	**Concentration (mg/L)**		25	50	75	100	125	0	Methanol control
	**Plants Emerged**	**7 d**	0.3 ± 0.6 ^1^	0	0	0	0	8	6
		**14 d**	0.3 ± 0.6	0.3 ± 0.6	1.0 ± 1.7	0.3 ± 0.6	0	10	5
**Lincomycin**	**Concentration (mg/L)**		6	12	18	24	30	0	Methanol control
	**Plants Emerged**	**7 d**	9.0 ± 1.0	8.0 ± 2.0	6.7 ± 2.1	6.7 ± 1.5	7.3 ± 0.6	8	/
		**14 d**	7.7 ± 2.1	8.0 ± 2.6	5.3 ± 3.5	6.3 ± 0.6	7 ± 1.0	10	/
**Roxarsone**	**Concentration (mg/L)**		5	25	45	65	80	0	Methanol control
	**Plants Emerged**	**7 d**	9.3 ± 0.6	8.0 ± 1.7	9.7 ± 0.6	9.0 ± 1.0	6.3 ± 0.6	8	/
		**14 d**	6.0 ± 3.0 *	4.3 ± 1.5 *	3.3 ± 2.3 *	2.7 ± 2.1 *	1.7 ± 0.6 *	10	/

^1^ data represents mean ± standard deviations, n = 3, the same below. * represents significant difference at *p* < 0.05 compared with the control group.

**Table 5:** Effect of lincomycin and roxsarsone on *A. thaliana* height and biomass (14 d).

**Lincomycin**	**Concentration (mg/L)**	6	12	18	24	30	0
	**Total Gross Dry Weight (g)**	0.0033 ± 0.0008	0.0046 ± 0.0012	0.0040 ± 0.0030	0.0031 ± 0.0011	0.0074 ± 0.0007	0.0045
	**Max. Plant Height (cm)**	2.5 ± 0.5	2.7 ± 1.0	3.0 ± 2.8	2.9 ± 1.4	4.2 ± 1.3	3.6
**Roxarsone**	**Concentration (mg/L)**	5	25	45	65	80	0
	**Total Gross Dry Weight (g)**	0.0038 ± 0.0045	0.0010 ± 0.0005	0.0011 ± 0.0012	0.0019 ± 0.0005	0.0005 ± 0.0004	0.0045
	**Max. Plant Height (cm)**	1.7 ± 0.3	0. 8± 0.3	1.0 ± 0.5	1.8 ± 1.0	0.4 ± 0.1	3.6

**Table 6:** Activity inhibition numbers of daphnia in monensin and roxarsone groups at 48 h (n = 8).

**Monensin**	**Concentration (mg/L)**	9.5	14.7	20.0	22.7	35.0	0
	**Daphnia inhibited**	0	1.0 ± 0	2.3 ± 0.6	4.0 ± 1.0	8.0 ± 0	0
**Roxarsone**	**Concentration (mg/L)**	250.0	268.9	289.3	311.1	334.7	0
	**Daphnia inhibited**	0	1.3 ± 0.6	3.3 ± 0.6	5.7 ± 0.6	8.0 ± 0	0

**Table 7:** Effect of potassium dichromate on zebrafish mortality (n = 8).

**Drug Concentration (mg/L)**	0	250	300	350	400	450
**Mortality**	0	0	2	4	6	7

**Table 8:** Effect of monensin and roxarsone on zebrafish mortality (n = 8).

**Monensin**	**Concentration (mg/L)**	4	4.34	4.7	5.1	5.53	0
	**Mortality**	0	1	4	6	8	0
**Roxarsone**	**Concentration (mg/L)**	400	423	447	473	500	0
	**Mortality**	0	1	3	6	8	0

**Table 9:** Effect of monensin and roxarsone on earthworm mortality (n = 10).

**Monensin**	**Dose (g/kg Dry Soil)**		CK ^1^	200	258	332	427	550
	**Average Mortality**	**7 d**	0	0	0	1	3	5
		**14 d**	0	0	2	4	7	10
**Roxarsone**	**Dose (g/kg Dry Soil)**		CK	4	5.03	6.33	7.95	10
	**Average Mortality**	**7 d**	0	0	0	2	5	10
		**14 d**	0	0	3	6	10	10

^1^ CK represents control check.

**Table 10:** Effect of monensin and roxarsone on quail mortality (5 male and 5 female).

**Monensin**	**Drug Dose (mg/kg)**		0	80	400	800	1100	1500
	**Mortality**	**Male**	0	0	1	3	3	5
		**Female**	0	0	2	2	3	4
**Roxarsone**	**Drug Dose (mg/kg)**		0	30	52.7	100	162.3	284.8
	**Mortality**	**Male**	0	0	1	2	4	5
		**Female**	0	0	1	2	3	5

**Table 11:** Summary of acute toxicity data and category of drugs.

Species	Roxarsone		Monensin		Lincomycin	
	Data	Category	Data	Category	Data	Category
*S. obliquus* EC_50_ (96 h) mg/L	13.15	low	1.085	medium	0.813	medium
*A. thaliana* EC_50_ (14 d) mg/kg dry soil	32.18	low	<25	/	35.40	low
*E. fetida* LC_50_ (14 d) g/kg dry soil	5.74	low	0.346	low	>15	low
*D. rerio* LC_50_ (96 h) mg/L	452.7	low	4.76	medium	>2800	low
*D. magna* EC_50_ (48 h) mg/L	292.6	low	21.1	low	>400	low
*C. coturnix* LD_50_ (7 d) mg/kg	103.9	medium	672.8	low	>2000	low

**Table 12:** Basic properties of the test soils.

Test Soil	pH	Organic Matter (g/kg)	Electrical Conductance (μS/cm)	Total P (g/kg)	NH_4_^+^-N (mg/kg)	Total K (g/kg)
Podzol soil	6.876	26.00	180.3	0.958	12.50	2.53
Laterite soil	6.710	3.62	58.5	0.070	7.13	11.40
Black soil	5.257	263.69	605.5	1.980	19.74	6.97

**Table 13:** Optimal soil/solution ratios and equilibrium time in sorption tests.

Soil	Lincomycin		Monensin		Roxarsone	
	Ratio	Time (h)	Ratio	Time (h)	Ratio	Time (h)
Podzol soil	50:1	24	100:1	48	50:1	24
Laterite soil	50:1	24	20:1	24	50:1	48
Black soil	50:1	24	50:1	24	50:1	48

**Table 14:** Extracted drug recovery in test soils (%).

Drug	Podzol Soil		Laterite Soil		Black Soil	
	Recovery	RSD	Recovery	RSD	Recovery	RSD
Lincomycin	73.2–97.4	0.801–3.10	71.7–108	1.21–4.83	78.1–98.6	0.344–2.12
Monensin	84.6–91.8	1.98–4.62	81.0–111	0.620–4.62	90.3–106	1.72–5.10
Roxarsone	89.9–92.6	1.15–3.18	91.2–99.3	1.34–1.69	79.4–96.7	1.36–3.87

## References

[1] Sarmah A.K., Meyer M.T., Boxall A.B. (2006). A global perspective on the use, sales, exposure pathways, occurrence, fate and effects of veterinary antibiotics (VAs) in the environment. Chemosphere.

[2] Mellon M., Benbrook C., Benbrook L.K. (2001). Hogging it: Estimates of Antimicrobial Abuse in Livestock.

[3] Kong W.D., Zhu Y.G., Liang Y.C. (2007). Uptake of oxytetracycline and its phytotoxicity to alfalfa (*Medicago sativa* L.). Environ. Pollut..

[4] Pan M., Chu L.M. (2017). Fate of antibiotics in soil and their uptake by edible crops. Sci. Total. Environ..

[5] Hurtado C., Dominguez C., Perez-Babace L. (2016). Estimate of uptake and translocation of emerging organic contaminants from irrigation water concentration in lettuce grown under controlled conditions. J. Hazard. Mater..

[6] Lee H.C., Chen C.M., Wei J.T. (2018). Analysis of veterinary drug residue monitoring results for commercial livestock products in Taiwan between 2011 and 2015. J. Food Drug Anal..

[7] Aus D.B.T., Weber F.-A., Bergmann A. (2016). Pharmaceuticals in the environment-Global occurrences and perspectives. Environ. Toxicol. Chem..

[8] Wei R., Ge F., Zhang L. (2016). Occurrence of 13 veterinary drugs in animal manure-amended soils in Eastern China. Chemosphere.

[9] Paltiel O., Fedorova G., Tadmor G. (2016). Human exposure to wastewater-derived pharmaceuticals in fresh produce: A randomized controlled trial focusing on carbamazepine. Environ. Sci. Technol..

[10] Chen W.R., Ding Y., Johnston C.T. (2010). Reaction of lincosamide antibiotics with manganese oxide in aqueous solution. Environ. Sci. Technol..

[11] Wang M., Zhang B., Wang J. (2018). Degradation of lincomycin in aqueous solution with hydrothermal treatment: Kinetics, pathway, and toxicity evaluation. Chem. Eng. J..

[12] Jwad S.M., Abbas B., Jaffat H.S. (2015). Study of the protective effect of vitamin C plus E on lincomycin-induced hepatotoxicity and nephrotoxicity. Res. J. Pharm. Technol..

[13] Migliore L., Civitareale C., Brambilla G. (1997). Toxicity of several important agricultural antibiotics to *Artemia*. Water Res..

[14] Hagenbuch I.M., Pinckney J.L. (2012). Toxic effect of the combined antibiotics ciprofloxacin, lincomycin, and tylosin on two species of marine diatoms. Water Res..

[15] Domínguez C., Flores C., Caixach J. (2014). Evaluation of antibiotic mobility in soil associated with swine-slurry soil amendment under cropping conditions. Environ. Sci. Pollut. Res..

[16] Arikan O.A., Mulbry W., Rice C. (2018). The fate and effect of monensin during anaerobic digestion of dairy manure under mesophilic conditions. PLoS ONE..

[17] Zizek S., Hrzenjak R., Kalcher G.T. (2011). Does monensin in chicken manure from poultry farms pose a threat to soil invertebrates?. Chemosphere.

[18] Storteboom H.N., Kim S.C., Doesken K.C. (2007). Response of antibiotics and resistance genes to high-intensity and low-intensity manure management. J. Environ. Qual..

[19] Sassman S.A., Lee L.S. (2010). Sorption and degradation in soils of veterinary ionophore antibiotics: Monensin and lasalocid. Environ. Toxicol. Chem..

[20] Wang Y. (2010). Study on the Ecotoxicological Effects of Monensin on Earthworms. Master’s Thesis.

[21] Capleton A.C., Courage C., Rumsby P. (2006). Prioritising veterinary medicines according to their potential indirect human exposure and toxicity profile. Toxicol. Lett..

[22] Garbarino J.R., Bednar A.J., Rutherford D.W. (2003). Environmental fate of roxarsone in poultry litter. Part, I. Degradation of roxarsone during composting. Environ. Sci. Technol..

[23] Li Y., Jiang M., Thunders M. (2018). Effect of enrofloxacin and roxarsone on CYP450s in pig. Res. Vet. Sci..

[24] Zhang Y.M. (2007). Ecotoxicological Study on the Residue of Roxarsone. Master’s Thesis.

[25] Makris K.C., Salazar J., Quazi S. (2008). Controlling the fate of roxarsone and inorganic arsenic in poultry litter. J. Environ. Qual..

[26] Brown B.L., Slaughter A.D., Schreiber M.E. (2005). Controls on roxarsone transport in agricultural watersheds. Appl. Geochem..

[27] Zhang W. (2018). A study on Ecological Toxicity and Environmental Behavior of Ethanamizuril. Master’s Thesis.

[28] Rutherford D.W., Bednar A.J., Garbarino J.R. (2003). Environmental fate of roxarsone in poultry liner. Part II. Mobility of arsenic in soils amended with chicken manure. Environ. Sci. Technol..

[29] Jiang C.A., Zhai X.F., Wang Y. (2013). Adsorption of ascorbic acid and roxarsone additives in different soils. J. South. China Agric. Univ..

[30] (2014). National Standards of People’s Republic of China. GB/T31270-2014 (Test Guidelines on Environmental Safety Assessment for Chemical Pesticides).

[31] Petrovic A.M., Larsson-Kovach I. (1996). Effect of maturing turfgrass soils on the leaching of the herbicide mecoprop. Chemosphere.

[32] Fiolka M.J. (2012). Activity and immunodetection of lysozyme in earthworm *Dendrobaena veneta* (Annelida). J. Invertebr. Pathol..

[33] Singh R.P., Srivastava G. (2009). Adsorption and movement of carbofuran in four different soils varying in physical and chemical properties. Adsorpt. Sci. Technol..

[34] Duan Y., Bao N., Li J. (2018). Fate characteristics of florasulam in soil environment. Guizhou Agric. Sci..

[35] Zhang X.H., Wan T., Cheng W. (2018). Effects of quinolones and sulfonamides on the growth of green algae. J. Water Resour. Water Engin..

[36] Peng Q.C., Song J.M., Li X.G. (2001). Biogeochemical characteristics and ecological risk assessment of pharmaceutically active compounds (PhACs) in the surface seawaters of Jiaozhou Bay, North China. Environ. Pollut..

[37] Bergmann T. (2002). Synergy of light and nutrients on the photosynthetic efficiency of phytoplankton populations from the Neuse River Estuary, North Carolina. J. Plankton Res..

[38] Hoagand R.E. (1996). Herbicidal properties of the antibiotic monensin. J. Sci.Food Agric..

[39] (2011). National standards of People’s Republic of China. GB/T 27861-2011 (Chemicals-Fish Acute Toxicity Test).

[40] Liu P., Wang M.Z., Zhong W.X. (2018). Stress-responsive genes (*hsp70* and *mt*) and genotoxicity elicited by roxarsone exposure in *Carassius auratus*. Environ. Toxicol. Pharmacol..

[41] Cai M.T., Hou G.Q., Xi H. (2018). Combined toxicity of co-exposure of typical antibiotic and heavy metal copper on freshwater green algae and zebrafish. J. Zhejiang Shuren Univ..

[42] Wenshyg C., Kuolung C., Bi Y. (1997). Effects of roxarsone on performance, toxicity, tissue accumulation and residue of eggs and excreta in laying hens. J. Sci. Food Agric..

[43] Otvos L. (2005). Antibacterial peptides and proteins with multiple cellular targets. J. Pept Sci..

[44] Wang F.M., Chen Z.L., Zhang L. (2006). Arsenic uptake and accumulation in rice (*Oryza sativa* L.) at different growth stages following soil incorporation of roxarsone and arsanilic acid. Plant. Soil..

[45] Tasho R.P., Cho J.Y. (2016). Veterinary antibiotics in animal waste, its distribution in soil and uptake by plants: A review. Sci. Total Environ..

[46] (2008). National standards of People’s Republic of China. GB/T 21851-2008 (Chemicals-Adsorption-desorption Using a Batch Equilibrium Method), GB/T 21805-2008 (Chemicals-Alga Growth Inhibition Test), GB/T 21830-2008 (Chemicals-Daphnia sp., Acute Immobilisation Test).

[47] (1984). OECD Guidelines for the Testing of Chemicals, No.207 “Earthworm, Acute Toxicity Tests”.

[48] Helling C.S., Turner B.C. (1968). Pesticide mobility: Determination by soil thin-layer chromatography. Science.

[49] Yan J. (1994). Determination of the content of lincomycin hydrochloride injection by spectrophotometry. Shanghai Med. Pharm. J..

[50] Zeng Z.G., Liu B., Chen Y.H. (2007). Determination of monensin by spectrophotometry. Chin. J. Vet. Drug.

[51] Zhang F.F., Wang W., Yuan S.J. (2014). Biodegradation and speciation of roxarsone in an anaerobic granular sludge system and its impacts. J. Hazard. Mater..

[52] Deng X.R., Qian Q.M., Sun C. (2014). Comparative study on the toxic effects of lindane and chlorpyrifos on freshwater algae. Ecol. Environ..

